# Accuracy of ultrasonography for the diagnosis of pneumoperitoneum

**DOI:** 10.1186/2036-7902-7-S1-A14

**Published:** 2015-03-09

**Authors:** P Nazerian, C Tozzetti, A Petrioli, M Ottaviani, F Trausi, M Baioni, S Grifoni

**Affiliations:** 1 Department of Emergency Medicine and Radiology Department, Careggi University Hospital, Firenze, Italy

## Background

Clinical signs and symptoms of pneumoperitoneum are not specific and abdomen radiography is positive in less than 50% of cases. Ultrasonography (US) accuracy for the diagnosis of pneuperitoneum is still unknown.

## Objective

1) define the accuracy of abdominal US for the diagnosis of pneumoperitoneum; 2) define the accuracy of a “2 scan-fast exam” vs a full abdominal exam; 3) compare accuracy of US and abdomen radiography

## Patients and methods

Study patients: 11 consecutive adults with a diagnosis of pneumoperitoneum by CT. Control patients: 11 consecutive adults with severe acute abdominal pain with a diagnosis other than pneumoperitoneum by CT. US examination has been performed with a convex and a linear probe using the following scans: epigastrium, right and left hypocondrium, umbelical area and right hypocondrium. All exams were recorded in a video of 5 seconds and each videos reviewed by 2 radiologists and 2 senior physicians blinded to other imaging studies. The reviewers fulfilled a standardized form signing for each scan either presence or absence of pneumoperitonem signs (enhancement of the peritoneum stripe with ring-down artifacts or “comet tails” starting from peritoneum). If one of the signs of pneumopeitoneum was present in at least one scan, the patient was considered to have a US diagnosis of pneumoperitoneum. The reviewers also evaluated abdomen radiography for the presence/absence of pneumoperitoneum. CT was considered the gold standard.

## Results

1) Accuracy of abdomen US was 88.6%. Sensitivities of convex and linear probes were similar (88.6% vs 84.1%), while specificity of convex was lower than linear probe (81.8% vs 95.5%). 2) Accuracy of a “2 scan-fast exam” was similar to global exam (87.5%). 3) Abdominal radiography sensitivity (72.2%) was lower than US while specificity (92.5%) was higher.

## Conclusions

Abdominal US has a good accuracy. The 2 scan-fast exam has a similar accuracy of the full abdominal exam. US sensitivity is superior to abdominal radiography thus ultrasonography can be a useful imaging modality for the detection of pneumoperitoneum.

